# Bouveret’s Syndrome: A Diagnostic and Therapeutic Approach to an Unusual Complication of Cholelithiasis

**DOI:** 10.7759/cureus.71046

**Published:** 2024-10-07

**Authors:** Nora Lis Flores-Olmos, Francisco Javier Hernández Álvarez, Daniel Alejandro Gamón Briseño, Rocio del Carmen Prieto Ramos, Ricardo Frausto Luján

**Affiliations:** 1 Department of General Surgery, Hospital Regional "Dr. Valentín Gómez Farías" ISSSTE, Zapopan, MEX; 2 Department of Surgery, General Hospital "Dr. Aurelio Valdivieso", Oaxaca, MEX; 3 Department of General Surgery, Hospital General ISSSTE Zacatecas, Zacatecas, MEX; 4 Department of Cardiology, Hospital Regional "Dr. Valentín Gómez Farías" ISSSTE, Zapopan, MEX

**Keywords:** biliary fistula, biliary ileus, bouveret syndrome, cholelithiasis, gastric obstruction

## Abstract

Bouveret's syndrome is a rare form of bowel obstruction caused by the impaction of a large gallstone through a cholecystoduodenal fistula, leading to gastric outlet obstruction. This article aims to highlight the clinical presentation and management of this syndrome, given its low incidence and high mortality rate of 12% to 30%. We present the case of an 83-year-old patient with a history of diabetes and hypertension who presented with abdominal pain, distension, and vomiting. Computed tomography revealed duodenal obstruction due to a large gallstone. A diagnostic laparotomy was performed, allowing for the removal of the stone through enterotomy. The patient had an uncomplicated recovery and was discharged on the seventh day of hospitalization. Early diagnosis and effective management are crucial, with endoscopy as the preferred initial treatment, although surgery may be necessary if endoscopy fails. Tailoring the treatment to the patient's condition is essential to improving outcomes and reducing associated mortality.

## Introduction

Complications associated with cholelithiasis are common and include acute cholecystitis, choledocholithiasis, pancreatitis, obstructive jaundice, mucocele and empyema of the gallbladder, and bowel obstruction [1]. Bouveret's syndrome is an uncommon form of bowel obstruction caused by the passage and impaction of a large gallstone through a cholecystoduodenal fistula into the duodenum, resulting in gastric outlet obstruction [2,3]. The formation of the fistula is caused by chronic inflammation, which increases intraluminal pressure, leading to ischemia and perforation of the wall, allowing the gallstone to pass into the intestine [4].

This condition was first described by Dr. Erasmus Bartholin in 1654, who noted that gallstones could cause gastrointestinal obstruction through a biliary-enteric fistula, with the terminal ileum being the most common site of obstruction. However, Beaussier was the first to describe Bouveret's syndrome in 1770 as an entity encompassing bowel obstruction with the impaction of gallstones in the pylorus or duodenum, leading to gastric outlet obstruction. In 1841, Bonnet described this condition in two patients at autopsy. In 1896, Leon August Bouveret was the first to preoperatively diagnose and publish two case reports of Bouveret's syndrome [5,6].

## Case presentation

We present the case of an 83-year-old male patient with a history of type 2 diabetes mellitus and long-standing systemic hypertension who also has a pacemaker and no other previous surgical history. He began experiencing symptoms four days prior to hospitalization, characterized by generalized abdominal pain, predominantly epigastric, described as oppressive, accompanied by abdominal distension, and intolerance to oral intake due to nausea and vomiting of gastric contents, which led him to seek emergency care.

On initial physical examination, the patient exhibited signs of dehydration in the oral cavity and significant abdominal distension with decreased peristalsis. Upon palpation, the abdomen was soft, and compressible, with moderate deep tenderness in the epigastric region, and tympanic on percussion. No signs of peritoneal irritation were noted.

**Figure 1:**
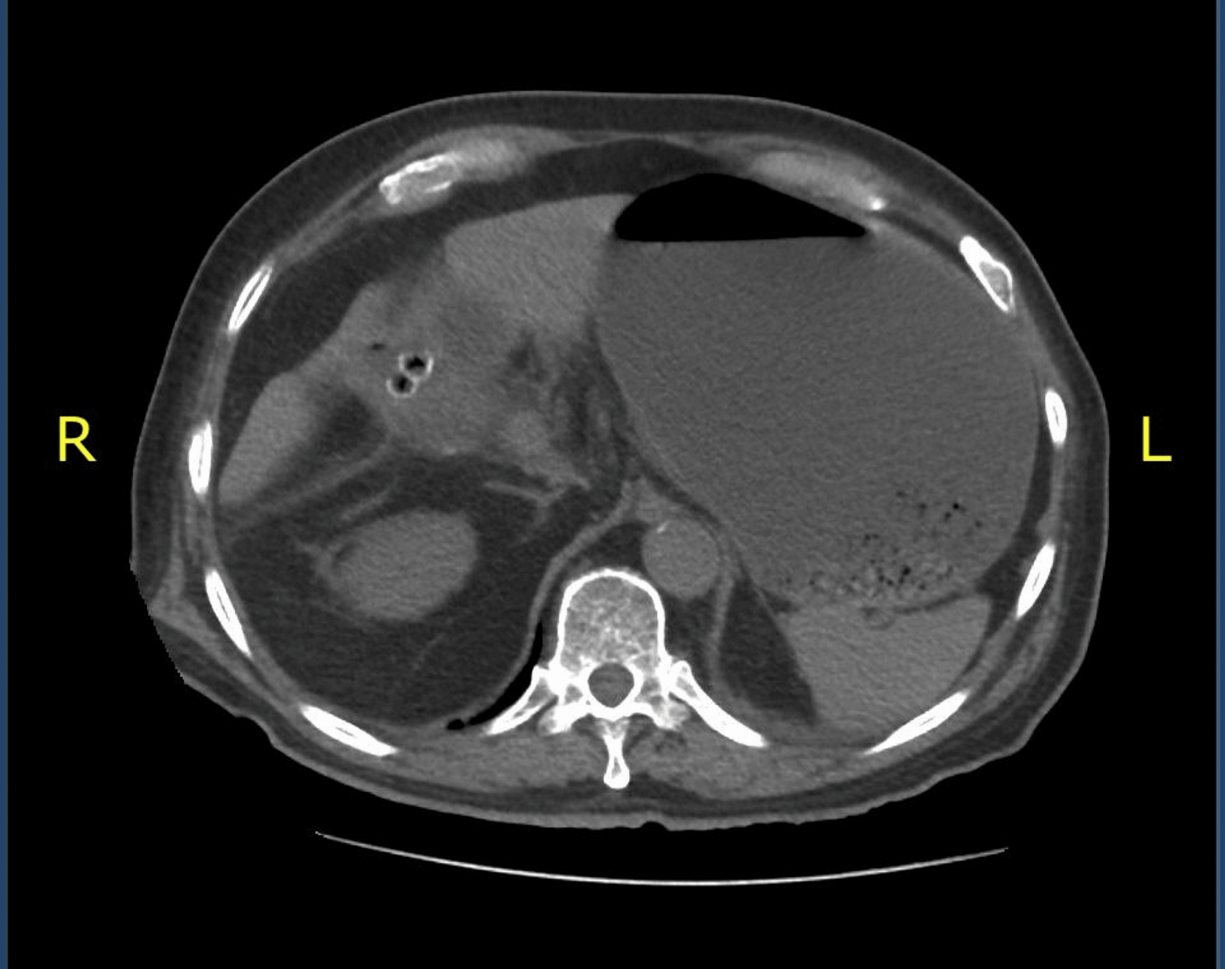
Presence of an image representing a fistulous tract between the gallbladder and the first portion of the duodenum; this tract measures approximately 20 mm.

**Figure 2:**
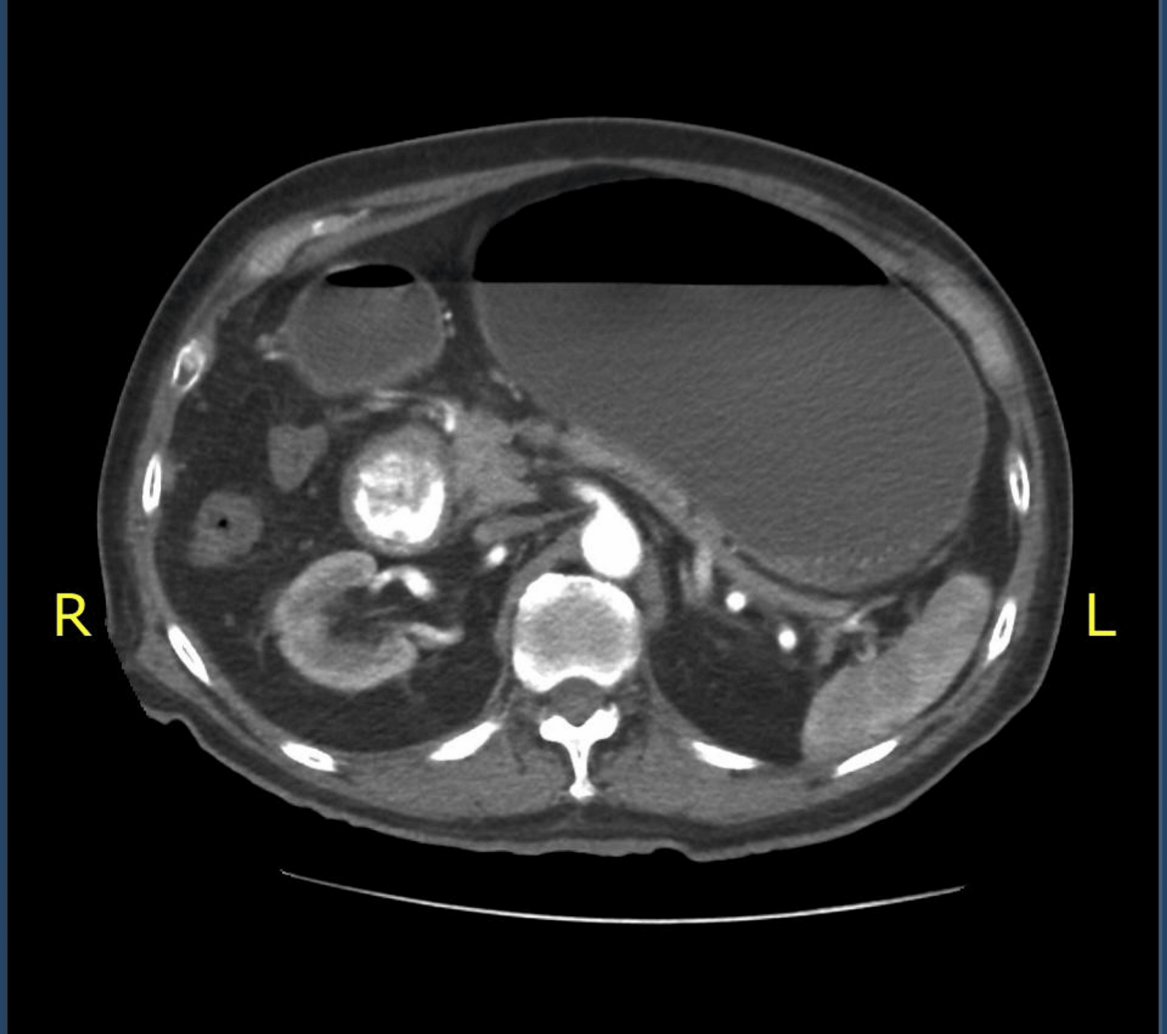
Duodenal obstruction in the second portion due to an ovoid, heterogeneous, predominantly hyperdense image with calcific origin, suggesting a gallstone, which measures approximately 40 mm in diameter.

**Figure 3:**
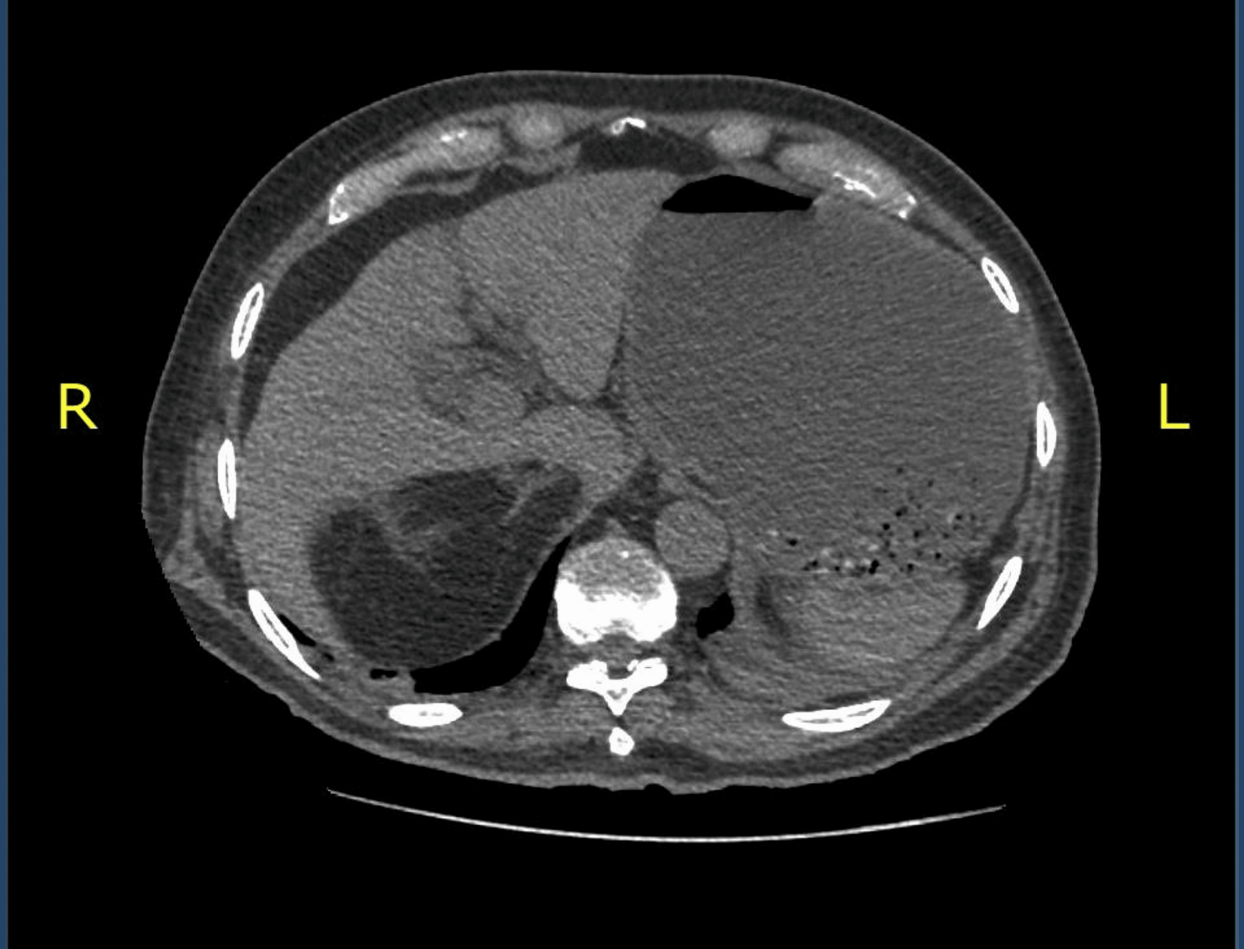
Gallbladder showing at least two images representing stones smaller than 10 mm in diameter with hyperdense sediment.

**Figure 4:**
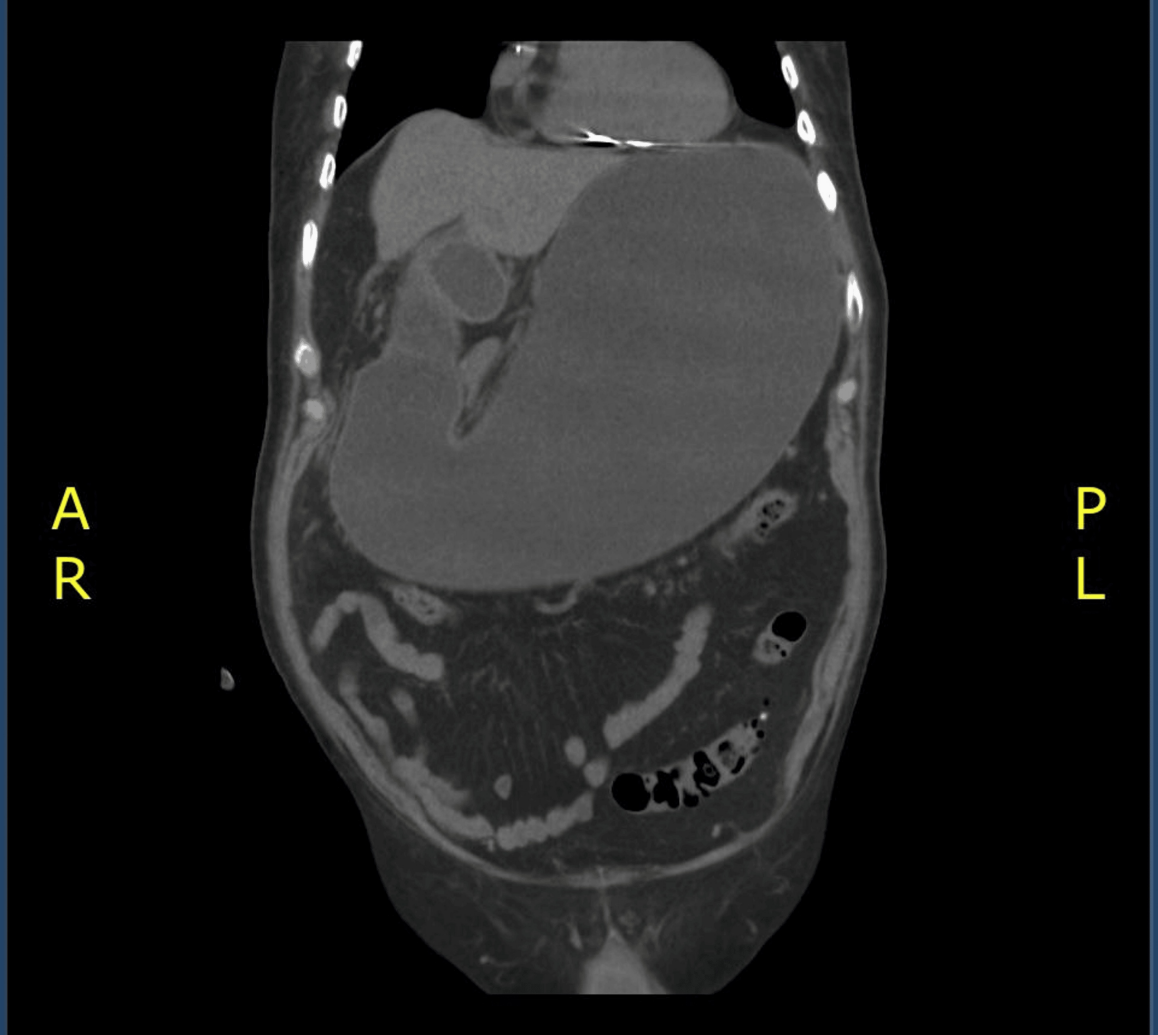
Significant dilation of the gastric chamber extending to the antrum and partially into the duodenal bulb; these changes are associated with the presence of duodenal obstruction.

In the emergency department, the patient was initially managed with a nasogastric tube, analgesics, and intravenous fluids, and a consultation with the general surgery service was requested. The patient was hemodynamically stable, with generalized and intense pain upon palpation in the epigastric region and no signs of peritoneal irritation at the time of examination. With no improvement from conservative management, a contrast-enhanced abdominal-pelvic CT scan was ordered, which revealed a cholecystoduodenal fistula at the level of the first portion of the duodenum; this tract measures approximately 20 mm (Figure 1), as well as duodenal obstruction in the second portion due to an ovoid, heterogeneous, predominantly hyperdense image with calcific origin, suggesting a gallstone, which measures approximately 40 mm in diameter (Figure 2). Gallbladder showing at least two images representing stones smaller than 10 mm in diameter with hyperdense sediment (Figure 3). Additionally, there was significant gastric dilation secondary to duodenal obstruction (Figure 4).

Laboratory results showed a glucose level of 119 mg/dL, blood urea nitrogen (BUN) of 14.8 mg/dL, and creatinine of 1.21 mg/dL. The total bilirubin was 0.61 mg/dL, with direct bilirubin at 0.18 mg/dL and indirect bilirubin at 0.43 mg/dL. Liver enzymes were reported as alanine aminotransferase (ALT) at 34 U/L and aspartate aminotransferase (AST) at 25 U/L. Lactate dehydrogenase (LDH) was elevated at 191 U/L. Electrolyte levels included calcium at 9.9 mg/dL, phosphorus at 2.7 mg/dL, chloride at 102 mEq/L, potassium at 4.1 mEq/L, sodium at 137 mEq/L, and magnesium at 2 mg/dL. Hematological parameters were as follows: red blood cells (RBC) at 5.02 x 10^6/μL, hemoglobin at 14.1 g/dL, hematocrit at 41.7%, platelets at 231 x 10^3/μL, and white blood cells (WBC) at 13.51 x 10^3/μL.

**Figure 5:**
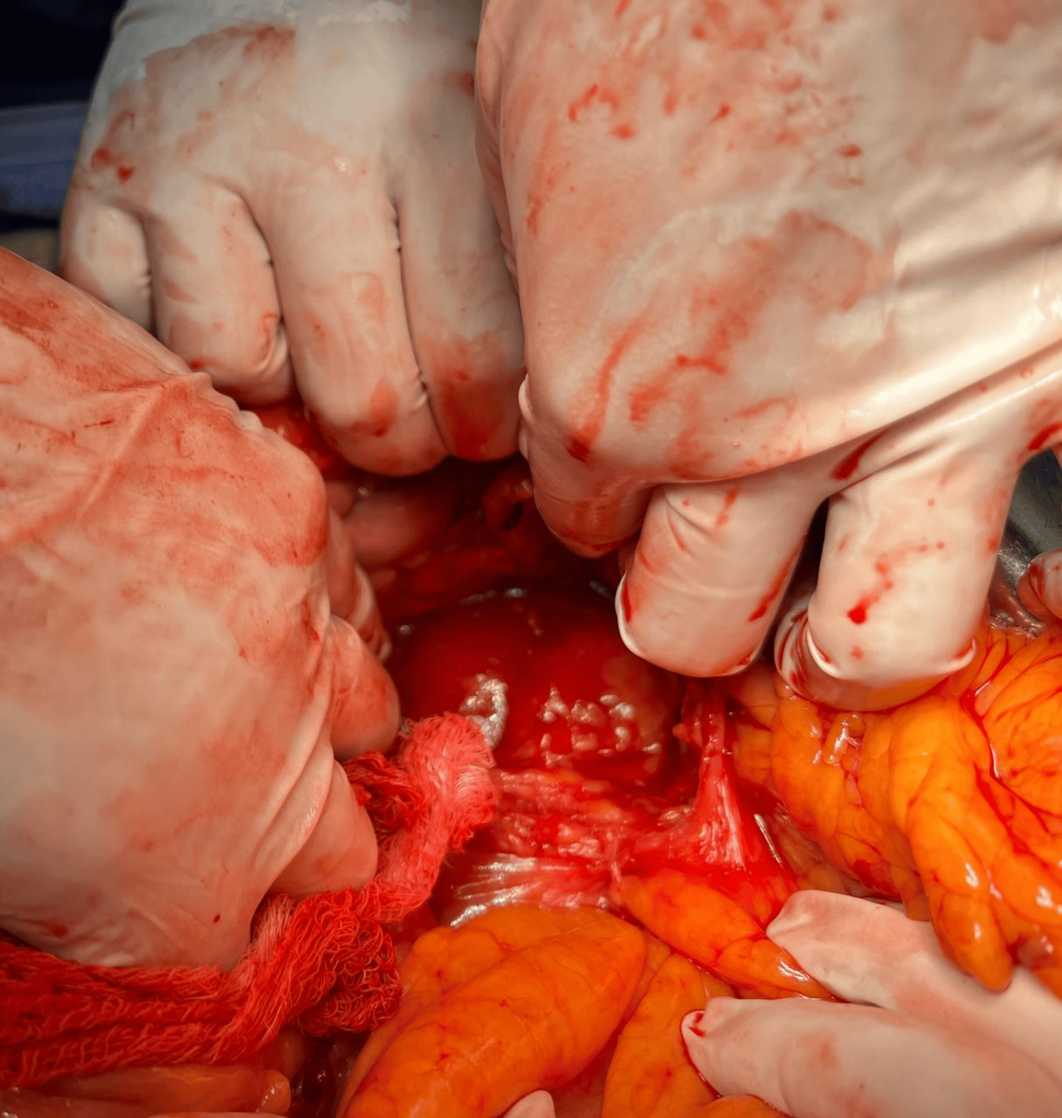
The fourth portion of the duodenum is observed with a stone lodged inside.

**Figure 6:**
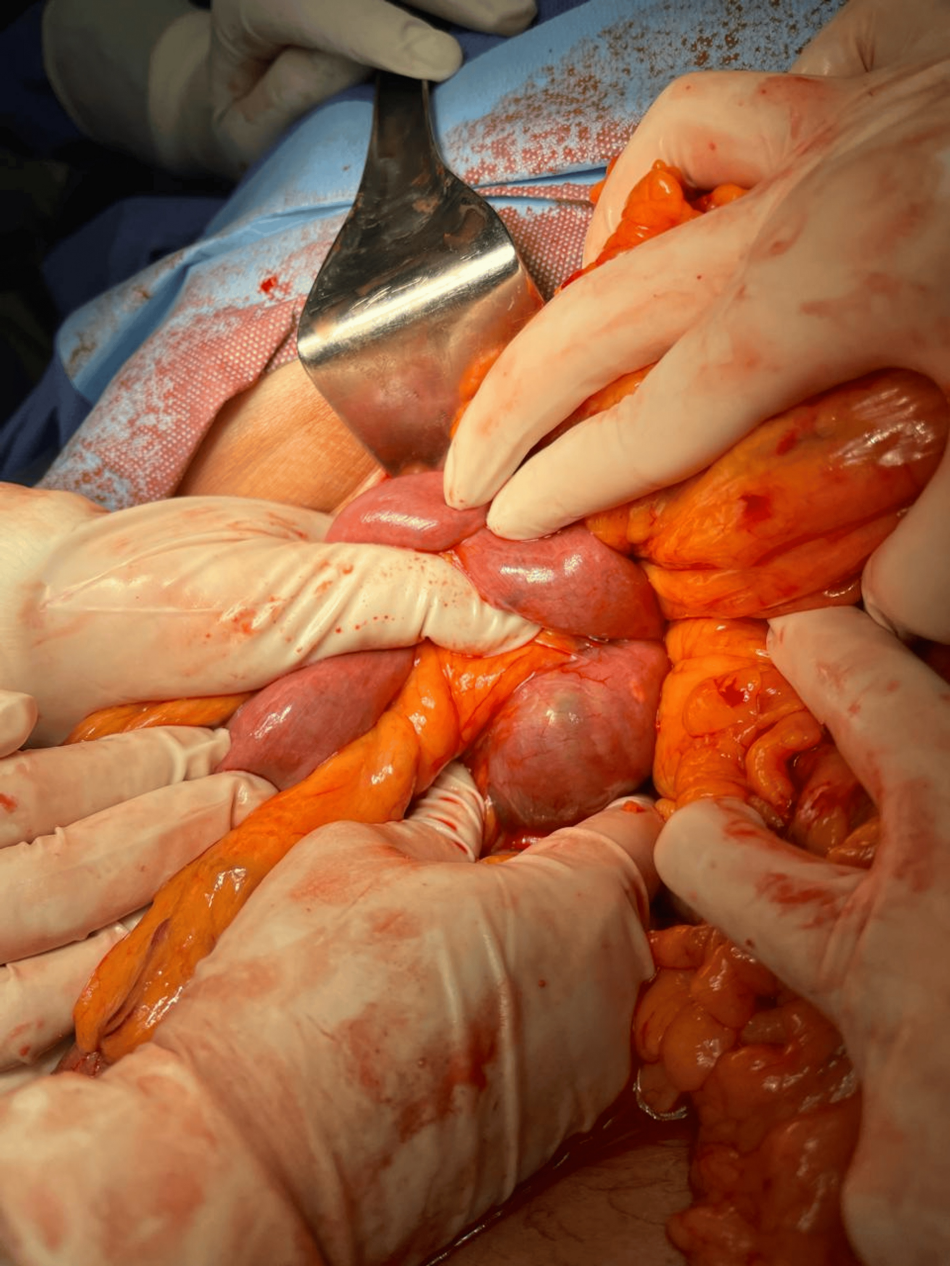
Stone lodged in the fourth portion of the duodenum after dissection of the Treitz ligament.

**Figure 7:**
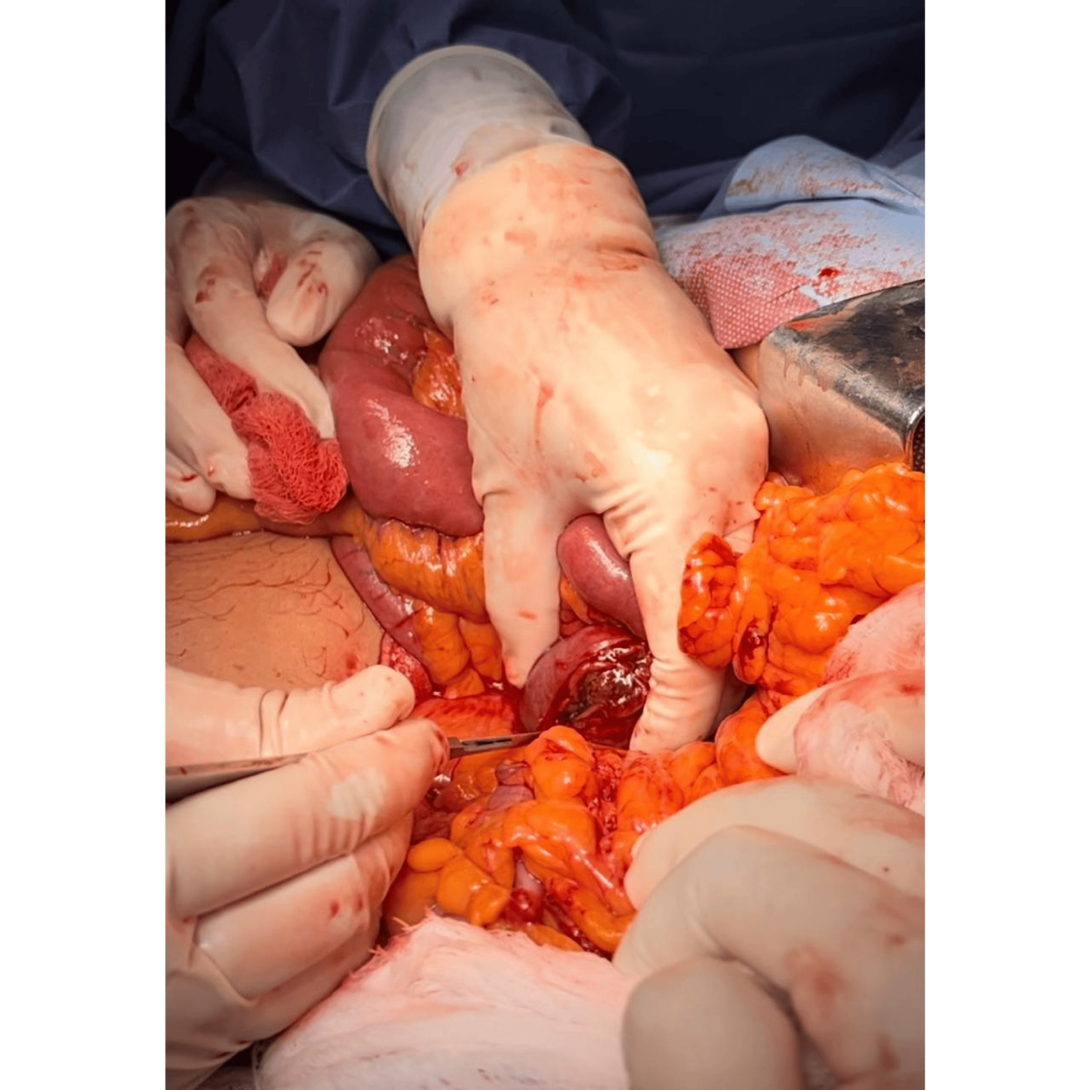
The enterotomy incision for the resection of the gallstone is observed.

**Figure 8:**
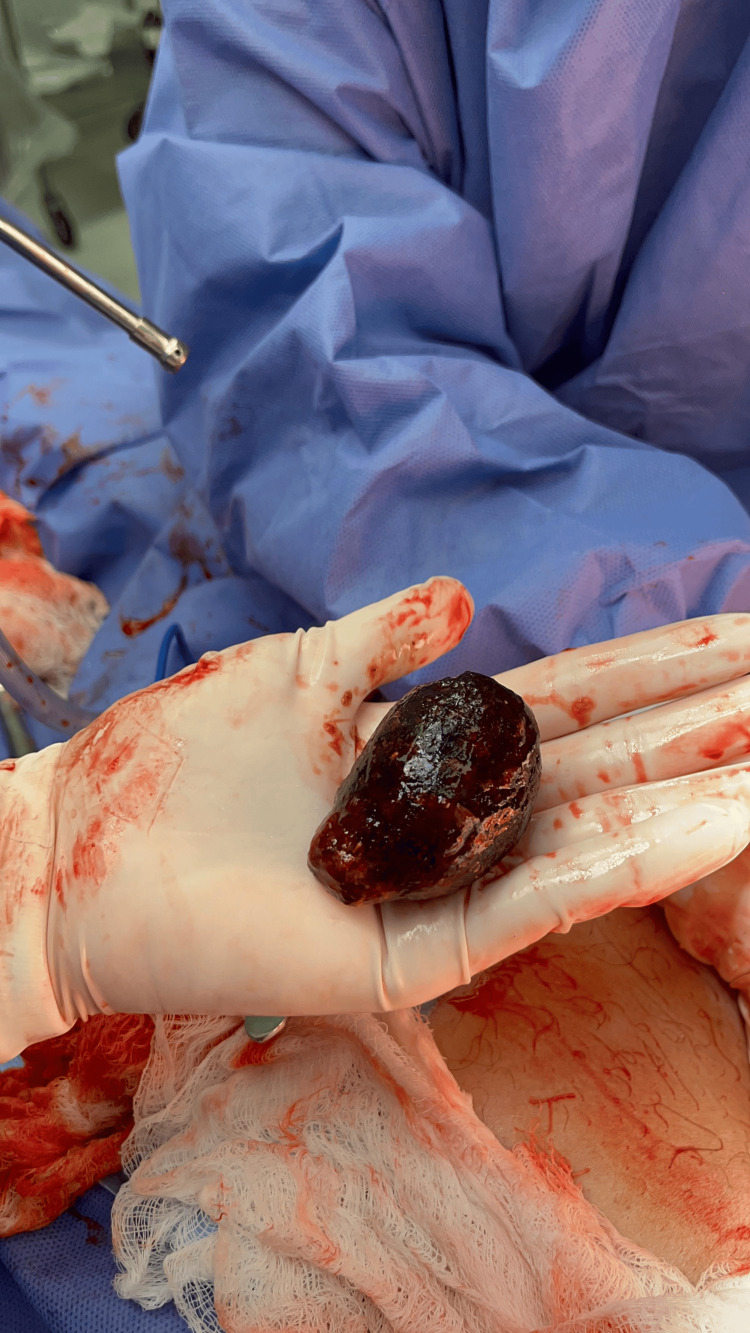
Excised gallstone.

The patient was taken to the operating room for diagnostic laparotomy, which revealed the following findings: a supra- and infraumbilical incision of approximately 25 cm was made, and dissection was carried out through the layers until the peritoneal cavity was accessed. A systematic examination of the bowel loops was performed, identifying a site of transition with obstruction caused by a gallstone approximately 4 x 6 cm in size located in the fourth portion of the duodenum (Figure 5). The Treitz ligament was dissected for better exposure (Figure 6), and an attempt to dislodge the stone was unsuccessful due to its size. An enterotomy was then performed at the site (Figure 7), the stone was extracted (Figure 8), and closure was achieved in two layers using PDS 1-0 Connell-Mayo sutures with invagination using Cushing stitches.

Postoperative management

The patient was kept nil per os (NPO) for 24 hours and subsequently started on a liquid diet, which was well tolerated and advanced the following day. The patient progressed without complications and was discharged on the seventh day with a follow-up arranged with the general surgery outpatient clinic.

## Discussion

Luminal complications of wandering gallstones occur in less than 1% of cholelithiasis cases and are classically presented as bowel obstruction. Bouveret’s syndrome comprises less than 5% of these cases, with approximately 300 observations reported in the literature to date [4]. It is crucial to recognize its characteristics and provide timely management, as it is associated with a mortality rate of 12% to 30% [7].

Risk factors and clinical presentation

Risk factors for Bouveret's syndrome include a history of cholelithiasis, with gallstones larger than 2.5 cm, which are more likely to become impacted and cause subsequent intestinal obstruction; the condition is more common in females with a ratio of 2:1 and typically occurs in the eighth decade of life [4,8].

Typical clinical signs on physical examination include abdominal pain, abdominal distension, and signs of dehydration due to recurrent vomiting. The most frequently identified presenting symptoms are nausea and vomiting (86%), abdominal pain (71%), and hematemesis (15%). Weight loss may occasionally be observed [5,9].

Hematemesis or melena can develop in cases of Bouveret's syndrome due to mucosal erosion caused by an impacted gallstone [10].

Diagnosis

In patients, metabolic alkalosis, hypokalemia secondary to intense vomiting, and elevated leukocyte and CRP levels due to the inflammatory process in the gallbladder, the fistula, and surrounding tissues can be observed [10].

Diagnosis can be made using abdominal X-ray, ultrasound, and CT. CT is considered the most accurate test, with 100% specificity; however, it may miss up to 25% of gallstones if they are radiolucent [9]. The Rigler triad (small bowel obstruction, pneumobilia, and an ectopic gallstone) is found on CT in approximately 78% of cases of bowel obstruction [7,10].

Isoattenuating gallstones, which are difficult to distinguish from surrounding bile, are visualized with magnetic resonance cholangiopancreatography in 15%-25% of cases and with endoscopy in 69% of cases [9].

Therapeutic approach

Untreated Bouveret's syndrome can lead to persistent gastric outlet obstruction, potentially causing anorexia, dehydration, malnutrition, and electrolyte imbalances. Intestinal perforation is the most dangerous consequence, as it can result in severe morbidities [6].

Bouveret's syndrome is a complex condition with a critical mortality rate ranging from 12% to 30%. This high mortality rate is attributed to the disease's complexity, nonspecific clinical presentation, and its tendency to affect elderly patients [7]. Furthermore, the management and diagnosis of Bouveret's syndrome are not standardized due to its rarity, with available options including endoscopy, laparoscopy, and open surgery [6].

Endoscopic treatment

Currently, endoscopic intervention represents the first-line treatment for Bouveret's syndrome. Endoscopic retrieval is a minimally invasive technique that may include mechanical lithotripsy, electrohydraulic lithotripsy, laser lithotripsy, and extracorporeal shock wave lithotripsy [8]. However, endoscopy has significant limitations. It does not allow for the closure of the fistula between the stomach/duodenum and the gallbladder or common bile duct, and fragmented stones may cause biliary ileus by obstructing distal parts of the intestine. Furthermore, the effectiveness of endoscopy largely depends on the operator's experience [10].

Surgical treatment

When endoscopic attempts fail or technical expertise is lacking, surgical treatment should be considered. The surgical strategy typically involves open gastrotomy, pylorotomy, or duodenotomy at or near the site of obstruction [11]. In cases where the duodenal gallstone can be maneuvered to the stomach, a gastrotomy may be sufficient for its removal. For a gallstone impacted in the distal duodenum or a gallstone that has migrated to the proximal jejunum, a distal enterotomy beyond the Treitz ligament can be utilized for stone extraction. The remainder of the small intestine should be examined to ensure no other large enteric stones that might cause postoperative obstruction [1]. There is controversy over whether cholecystectomy and fistula repair should be performed in a separate operation. Most patients, especially the elderly with multiple comorbidities, are not ideal candidates for extensive surgery in a single session. In such cases, a two-stage approach is recommended: stone extraction via gastrotomy or enterotomy in the first operation, and if the patient is fit for major surgery, cholecystectomy and repair of the fistula can be performed in a second surgical procedure. The incidence of biliary ileus before the second operation is about 5%, while cholangitis and cholecystitis are possible complications that should be monitored [10].

Recent developments and alternative options

Advancements in both surgical and endoscopic techniques are being made to improve the management of Bouveret's syndrome. For instance, electrohydraulic lithotripsy uses electrical energy to fragment obstructive stones within the duodenum, although this can potentially damage surrounding tissues. Research continues to refine these methods, enhance the efficiency of stone fragmentation, and minimize side effects [12]. Additionally, endoscopic retrieval using baskets and snares is an evolving technique, requiring specialized skills and advanced tools to increase success rates [10].

Another emerging strategy is percutaneous dissolution of gallstones, which utilizes topical medications to dissolve stones without the need for surgery. Although this method is still under investigation for Bouveret's syndrome, it presents a potential non-surgical option to address the underlying cause of the disease [6].

In summary, the management of Bouveret's syndrome should be tailored to the patient's medical conditions and specific clinical presentation. Options include endoscopic and surgical interventions, with the appropriate approach determined by the available technical expertise and the individual characteristics of the patient [1,13].

## Conclusions

Bouveret's syndrome, although a rare complication of cholelithiasis, presents a high mortality rate ranging from 12% to 30% due to its complexity and nonspecific clinical presentation. It constitutes less than 5% of cases of biliary ileus and predominantly affects elderly patients with a history of cholelithiasis and large stones. Diagnosis can be challenging, with computed tomography being the most accurate tool. Initial treatment is usually endoscopic, although this option has limitations. When endoscopy is ineffective or unavailable, surgery is necessary, often in a two-stage approach, particularly in patients with multiple comorbidities. Recent advances in endoscopic techniques and percutaneous stone dissolution could offer new treatment alternatives, though they are still under investigation. In conclusion, the management of Bouveret's syndrome should be tailored to the individual patient's conditions to optimize outcomes and reduce associated mortality.
